# The Effect of Variable Light Source and Light Intensity on the Growth of Three Algal Species

**DOI:** 10.3390/cells11081293

**Published:** 2022-04-11

**Authors:** Vitali Bialevich, Vilém Zachleder, Kateřina Bišová

**Affiliations:** Laboratory of Cell Cycles of Algae, Centre Algatech, Institute of Microbiology of the Czech Academy of Sciences, 37981 Třeboň, Czech Republic; bialevich@alga.cz (V.B.); zachleder@alga.cz (V.Z.)

**Keywords:** *Chlamydomonas reinhardtii*, *Parachlorella kessleri*, *Desmodesmus quadricauda*, light intensity, cell growth, LED, fluorescent tube

## Abstract

Light is the essential energy source for autotrophically growing organisms, including microalgae. Both light intensity and light quality affect cell growth and biomass composition. Here we used three green algae—*Chlamydomonas reinhardtii*, *Desmodesmus quadricauda*, and *Parachlorella kessleri*—to study the effects of different light intensities and light spectra on their growth. Cultures were grown at three different light intensities (100, 250, and 500 µmol m^−2^ s^−1^) and three different light sources: fluorescent lamps, RGB LEDs, and white LEDs. Cultures of *Desmodesmus quadricauda* and *Parachlorella kessleri* were saturated at 250 µmol m^−2^ s^−1^, and further increasing the light intensity did not improve their growth. *Chlamydomonas reinhardtii* cultures did not reach saturation under the conditions used. All species usually divide into more than two daughter cells by a mechanism called multiple fission. Increasing light intensity resulted in an increase in maximum cell size and division into more daughter cells. In *Parachlorella kessleri* cells, the concentration of photosynthetic pigments decreased with light intensity. Different light sources had no effect on algal growth or photosynthetic pigments. The results show a species-specific response of algae to light intensity and support the use of any white light source for their cultivation without negative effects on growth.

## 1. Introduction

Microalgae are prokaryotic or eukaryotic unicellular photoautotrophic organisms that live in a wide range of environmental conditions, not only in water but also on land [1]. The biomass of microalgae is a natural source of many biocompounds: chlorophyll is used in the food and cosmetic industries [2]; carotenoids, astaxanthin, antioxidants, and long-chain polyunsaturated fatty acids are used as dietary supplements in human nutrition and serve as feed for animals and fish [3]. Microalgae represent a promising alternative source of protein [4] and are considered a sustainable source for the production of biofuels such as bioethanol and biodiesel in the future [5,6].

The production of microalgal biomass and its biochemical content is species-dependent and influenced by many factors such as light, temperature, CO_2_ supply, culture medium composition, pH, bioreactor characteristics, etc. [7]. Among these factors, light is the essential factor for autotrophic growth of microalgae. Microalgae that grow with light use photosynthesis to convert light energy into chemical energy from molecular bonds and to sequester carbon from carbon dioxide in their biomass to support their growth and division. The energy accumulated in storage molecules during the light period is then used during the dark period [1,8,9]. In natural habitats, photoautotrophic growth of microalgae is supported by extremely variable natural light with its diurnal and seasonal variations. In addition, light penetration is influenced by geographic location and elevation as well as weather conditions. Similar constraints on light availability exist for large-scale algal cultivation in open systems, where uncontrolled natural light is used for microalgal cultivation. In contrast, small-scale algal cultivation, in photobioreactors, generally uses controlled artificial light. This allows control of factors such as the light intensity, the duration of the light period, and the spectral composition of the light [10,11,12]. In this way, light characteristics can be tailored to meet the specific requirements for biomass/biocomponent production of specific microalgal species.

Light intensity is a determining factor in microalgal growth, as photosynthetic rate correlates with light intensity [13]. The growth rate increases with light intensity up to a certain species-dependent level. Very high light intensities lead to photoinhibition [14], and very low light intensities below the saturation point limit the growth rate [15]. Moreover, light intensity affects not only biomass production but also its composition and plays a regulatory role in many biological processes [16]. Within the light spectrum, the wavelength range from 400 to 700 nm represents the photosynthetically active radiation (PAR), which is effective for photosynthesis [17]. Thus, the light spectrum is another factor affecting photoautotrophic growth [18]. Artificial light sources used for the cultivation of microalgae differ in the characteristics of the emitted light spectrum. Fluorescent lamps (especially cool fluorescent lamps with enhanced blue and red spectra), along with mercury and halogen lamps, have been widely used light sources for microalgae cultivation in the past. However, fluorescent lamps have several disadvantages: The emission spectrum does not match the selective absorption spectrum of aquatic plants (20–30% PAR efficiency); the light spectrum and intensity are not stable over time. Currently, light emitting diodes (LEDs) are considered the most efficient artificial light source with 80–100% PAR efficiency. LEDs have a narrow light spectrum and can specifically emit wavelengths corresponding to blue, green, yellow, orange, red, and other lights or their combinations, including a high fluence that mimics the spectrum of sunlight [7,19,20,21,22,23]. In addition, they have other advantages such as lower power consumption, long stability of light spectrum and intensity, and small size. Although LED light sources have higher initial costs, their operating costs and lifetime, together with their spectral advantages, make LEDs a more favorable economic choice compared with fluorescent lamps. 

The appearance of LEDs on the market has stimulated research on microalgae that use the narrow light spectrum to increase biomass productivity and change its composition [23]. Initial work compared the effects of growth of *Chlorella vulgaris* cultures grown with white and blue fluorescent light LED and showed better growth and higher lipid content in cultures grown with blue LEDs [17]. However, it is not clear whether the effect is related to the differences in the light source or in the light spectra of the two light sources. The growth rate, photosynthetic pigment content, fatty acid content, and fatty acid composition differed among cultures of *Chlorella vulgaris*, *Chlorella pyrenoidosa*, *Desmodesmus quadricauda*, and *Scenedesmus obliquus* grown under blue, red, and white LED light [24], with the highest biomass productivity obtained under blue light. This indicates a clear influence of light quality on algal growth and biomass composition. Similarly, *Chlorella* sp. cultures grown with blue light had higher lipid content than those grown under red light [25]. Blue light has been shown to lead to the formation of larger cells in both *Chlamydomonas reinhardtii* [26] and (*Para)Chlorella kessleri* [27]. This effect is clearly related to the light quality since in the first study filtered light from a tungsten halogen lamp was used, while in the second study the light source LED was used. Thus, despite the numerous studies that have been conducted using LEDs, it should not be forgotten that both the light source and the light color affect microalgal growth and/or biomass composition [23]. As LEDs are gradually replacing fluorescent tubes, it is of interest to investigate whether the different light sources with quite different spectra would affect the growth of the cultures. 

In this study, three model microalgae with different forms—*Chlamydomonas reinhardtii* (biflagellate with positive and negative phototaxis), *Desmodesmus quadricauda* (non-motile coenobium, consisting of 4- to 8-cylindrical cells), and *Parachlorella kessleri* (non-motile single spherical cells)—were grown under different white light sources—fluorescent lamps, RGB LED, and white LED—providing the same amount of photosynthetically active radiation (PAR) with different spectral composition. The three algal species were grown at different light intensities (100, 250, and 500 µmol m^−2^ s^−1^) for an extended period of time. In this way, the effects of light intensity, light source, and acclimation to it were studied simultaneously.

## 2. Materials and Methods

### 2.1. Microalgae Strains

*Chlamydomonas reinhardtii* (CC–1690, wild type) was obtained from Chlamydomonas Resource Center (St. Paul, MI, USA, http://www.chlamy.org, accessed on 3 January 2020), and it was grown in high salt (HS) mineral medium according to Sueoka [28] with slight modifications [29]. *Desmodesmus quadricauda* (originally *Scenedesmus quadricauda* (Turpin) Brébisson, strain Greifswald 15) and *Parachlorella kessleri* (strain CCALA 255) were both obtained from the Culture Collection of Autotrophic Organisms (CCALA) at the Institute of Botany, Czech Academy of Sciences (Třeboň, Czech Republic, https://ccala.butbn.cas.cz, accessed on 3 January 2020). The strains were grown in ½ ŠS medium [30]. All the strains were routinely sub-cultured every three weeks on the appropriate medium solidified by addition of agar (1.5%) and grown at 30 °C on light panels of fluorescent lamps at incident light intensity 100 µmol m^−2^ s^−1^ of photosynthetically active radiation (PAR).

### 2.2. Culture Conditions

**Figure 1:**
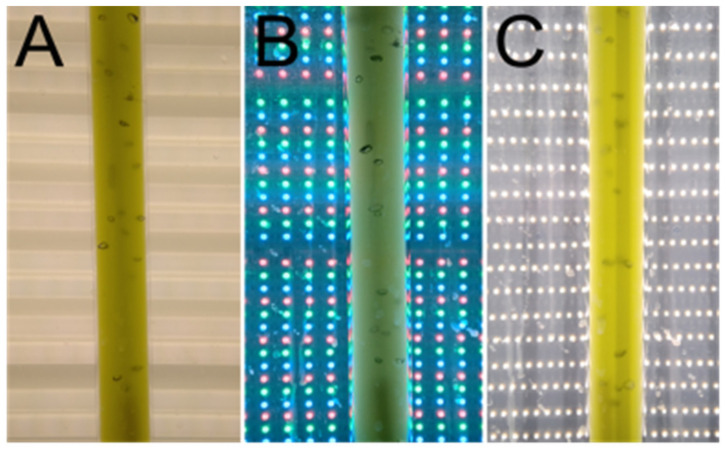
Light sources used for the cultivation. (**A**) Fluorescent lamps, (**B**) RGB LEDs, (**C**) white LEDs.

**Figure 2:**
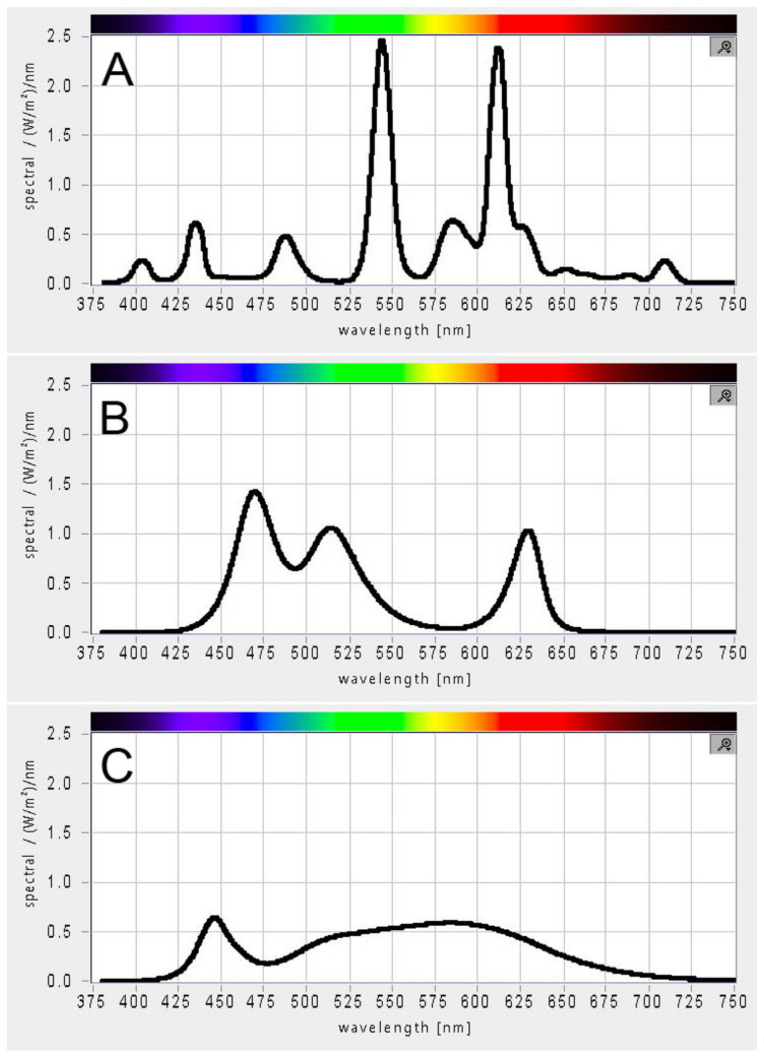
PAR spectrum of white light sources used for microalgae cultivation. (**A**) Fluorescent lamps, (**B**) RGB LEDs, (**C**) white LEDs.

For the inoculum, the algae were sub-cultured from the plates into 300 mL of liquid medium in glass cylinders (inner diameter 30 mm, height 500 mm). The cylinders were placed in water bath to maintain a constant temperature of 30 °C and “aerated” with a mixture of air and CO_2_ (2%, *v*/*v*) at a flow rate of 15 L h^−1^. The water bath containing cylinders was illuminated from one side by a panel of dimmable fluorescent lamps (OSRAM Dulux L, 55 W/040, Milano, Italy) with incident light intensity 200 µmol m^−2^ s^−1^ PAR (Figure 1A). The algae were grown for 2–3 days until the cell density reached about 1 × 10^6^ cell mL^−1^ and then were maintained at about this density by dilution every day. For the experiment, the cultures were diluted to the starting optical density (OD750) of approximately 0.1. The diluted culture was split into three replicates and each of them was grown at different source of light (fluorescent lamps, RGB LED, white LED) (Figure 1 and Figure 2). Every 24 h, new cultures were re-started by dilution from the cultures grown at the same light source. The cultures were grown at three different light intensities (100, 250, and 500 µmol m^−2^ s^−1^). All experiments were repeated three times.

### 2.3. Light Spectrum and Intensity Measurement

The light spectrum of each light source (Figure 2) was measured using BTS256-PAR light meter (Gigahertz-Optik, Tuerkenfeld, Germany). The incident light intensity at the surface of the vessel was measured using Walz ULM-500 light meter equipped with flat sensor (Walz, Effeltrich, Germany).

### 2.4. Quantum Yield Measurement

Aliquots of 2 mL were withdrawn from the culture and dark adapted in 10 × 10 mm plastic cuvettes for 30 min. Quantum yield was measured using an AquaPen-C AP-C 100 (Photon Systems Instruments, Drasov, Czech Republic).

### 2.5. Cell Volume and Number

Cell volume and number were measured using a Beckman Coulter Multisizer 4 (Beckman Coulter Life Sciences, Indianapolis, IN, USA) by diluting 50 µL of fixed (0.2% glutaraldehyde) cell suspension into 10 mL of 0.9% NaCl (*w*/*v*) electrolyte solution.

### 2.6. Cell Doubling Time

Cell doubling time (*Td*) was calculated based on cell counts using the formula:

(1) $$Td=\left(t_{x}-t_{0}\right)\frac{\ln\left(2\right)}{\ln\frac{N_{x}}{N_{0}}}$$

where *N*_0_ is the cell number at sampling time at 0 h (*t_0_*), and *N_x_* is the cell number at the sampling time 23 h (*t_x_*) of the experiment [31]. The data are expressed in hours.

### 2.7. Photosynthetic Pigment Contents

Photosynthetic pigments were analyzed as described previously [32]. Briefly, cell pellets were re-suspended in of phosphate buffer (pH 7.7) containing a pinch of MgCO_3_ and stored at −20 °C until used. Cells were disintegrated by vortexing for 10 min in the presence of zirconium beads, and the chlorophylls were extracted with 100% acetone followed by another extraction with 80% (*v*/*v*) acetone. The combined extract was read at 750, 664, 647, 470, 450 nm, and the chlorophyll content was calculated using the method from MacKinney [33]. The following equations were used for chlorophyll a (chl a) (12.25 × A664 nm − 2.79 × A647 nm), chlorophyll b (chl b) (21.5 × A647 nm − 5.1 × A664 nm), and carotenoids ((1000 × A470 nm) − (1.82 × chl a) − (85.02 × chl b))/198) [33,34,35].

### 2.8. Statistical Analysis

Statistical analyses, including the basic ones, were carried out using MS Excel 2010 (Microsoft Corporation, Redmond, WA, USA) using Real Statistics Resource Pack (https://www.real-statistics.com/free-download/real-statistics-resource-pack/, accessed on 27 July 2021). Two-way ANOVA followed by Tukey’s HSD test was used to compare maximum biomass, maximum cell size, and cell doubling time. A *p*-value < 0.05 was considered significant.

## 3. Results

Growth of three microalgae species *C. reinhardtii*, *D. quadricauda,* and *P. kessleri* was monitored when grown under three white light sources: fluorescent lamps, RGB LEDs, and white LEDs. To all the cultures, the same molar concentration of photons in a form of the same incident light intensity was provided. However, given the difference in the light spectrum supplied by each of the light source (Figure 2), the proportions of photons with different energies varied among the light sources. The experiments followed two basic rationales: (1) to detect possible effects of light source on growth rate and (2) to compare light intensity reactions among the three species. To address the former rationale, the algal cultures were grown on the different light source for three successive generations with dilution after each generation. To address the latter rationale, the cultures were grown at three different incident light intensities: 100, 250, and 500 µmol photons m^−2^ s^−1^ of PAR.

### 3.1. Maximum Potential Quantum Efficiency of Photosystem II

**Figure 3:**
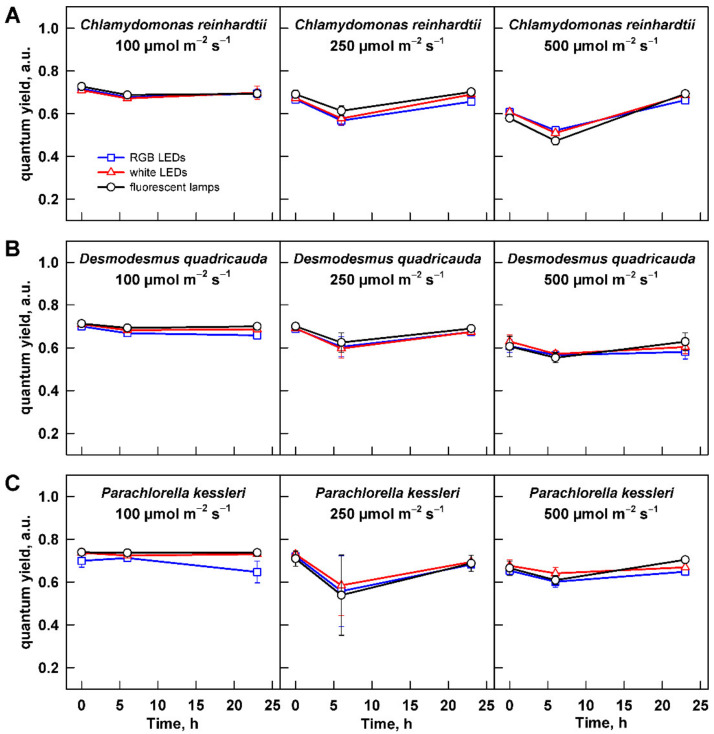
Quantum yield of the cultures grown at different light intensities. Cultures of (**A**) *Chlamydomonas reinhardtii*, (**B**) *Desmodesmus quadricauda*, and (**C**) *Parachlorella kessleri* were grown at incident light intensity 100, 250, and 500 µmol m^−2^ s^−1^ on different light sources: fluorescent lamps (black circles), RGB LEDs (blue squares), and white LEDs (red triangles). Data of three biological replicates are presented as means ± SE.

Firstly, the potential toxic effect of any of the light sources, light intensity, or their combination was assessed using quantum yield (QY), which is a measure of maximum potential quantum efficiency of Photosystem II and is widely used as a photosynthesis stress indicator. All the values were within the physiological values, confirming no damaging effect of any light source or light intensity (Figure 3). Values of QY measured for all the organisms at low light intensity were stable throughout the cultivation. For the two higher light intensities—250 and 500 µmol m^−2^ s^−1^—there was a slight drop in QY at 6 h of cultivation. This was statistically significant only for *C. reinhardtii* (two-way ANOVA, Tukey’s HSD test, *p* < 0.05, *n* = 3) but not for the other two organisms. This might have reflected the reaction of the cells to dilution from higher to lower cell and biomass density, which increased mean light intensity available to them. *C. reinhardtii* also showed statistically significantly different values of QY between the light intensities (two-way ANOVA, Tukey´s HSD test, *p* < 0.05, *n* = 3). At the end of cultivation, the QY of all organisms recovered to the starting values (Figure 3A–C). 

### 3.2. Effect of Light Intensity

Each autotrophic organism has a certain range of light intensities at which it thrives. The light intensity below the lower threshold does not allow for an efficient growth, while light intensity above the upper threshold will damage the cell and slow or even prevent further growth. Saturation light intensity is a light intensity that will lead to the maximum growth rates; increasing light intensity above this value will not significantly increase growth rates. The saturation light intensity is dependent on photosynthesis saturation but is also affected by cell concentration, cell shape, and cell shading.

#### 3.2.1. Cell Growth

**Figure 4:**
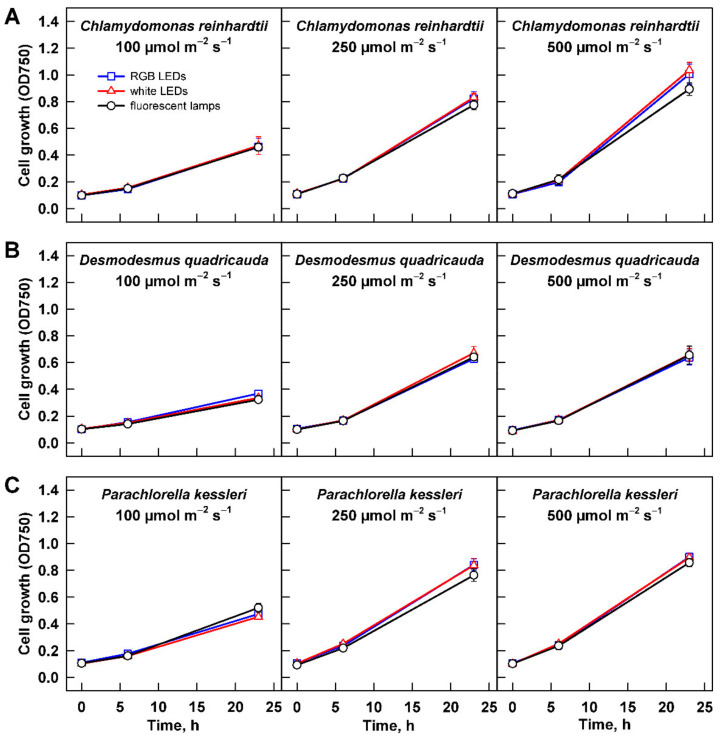
Growth of the cultures cultivated at different light intensities. Cultures of (**A**) *Chlamydomonas reinhardtii*, (**B**) *Desmodesmus quadricauda*, and (**C**) *Parachlorella kessleri* were grown at incident light intensity 100, 250, and 500 µmol m^−2^ s^−1^ on different light sources: fluorescent lamps (black circles), RGB LEDs (blue squares), and white LEDs (red triangles). Cell growth is expressed as optical density at 750 nm. Data of three biological replicates are presented as means ± SE.

Growth of the three algal species at increasing light intensities revealed species-specific response to the changing conditions. For all the organisms, the slowest growth was observed at 100 µmol m^−2^ s^−1^ (Figure 4). With increasing light intensity, the behavior of each species differed. Growth rate of *C. reinhardtii* cultures increased at 250 µmol m^−2^ s^−1^, and it was improved further at 500 µmol m^−2^ s^−1^ (Figure 4A). Under the growth conditions tested, the cultures did not reach a saturation. When the changes in growth were expressed by slopes of the corresponding curves, there was statistically significant difference among all three treatments (two-way ANOVA, *p* < 0.05, *n* = 3). Similarly, there was a statistically significant difference among the values of maximum optical density (Table 1). The value reached at 100 µmol m^−2^ s^−1^ increased about 1.5 fold at 250 µmol m^−2^ s^−1^, and it increased further by about 30% at 500 µmol m^−2^ s^−1^. The less prominent growth increase between the two latter intensities suggested that at 500 µmol m^−2^ s^−1^ the culture was nearing its light saturation intensity. Cultures of *P. kessleri* and *D. quadricauda* behaved differently than *C. reinhardtii* as they reached the saturation already at 250 µmol m^−2^ s^−1^, and the growth did not further improve by cultivation at 500 µmol m^−2^ s^−1^ (Figure 4, compare B and C with A). This was also reflected by the maximum optical density reached by the two organisms (Table 1). Interestingly, the increase in both growth rate and maximum optical density was more prominent in the two organisms compared with *C. reinhardtii* (Figure 4, Table 1). Of the three organisms, *D. quadricauda* was the slowest grower (Figure 4, Table 1).

#### 3.2.2. Cell Size and Number

**Figure 5:**
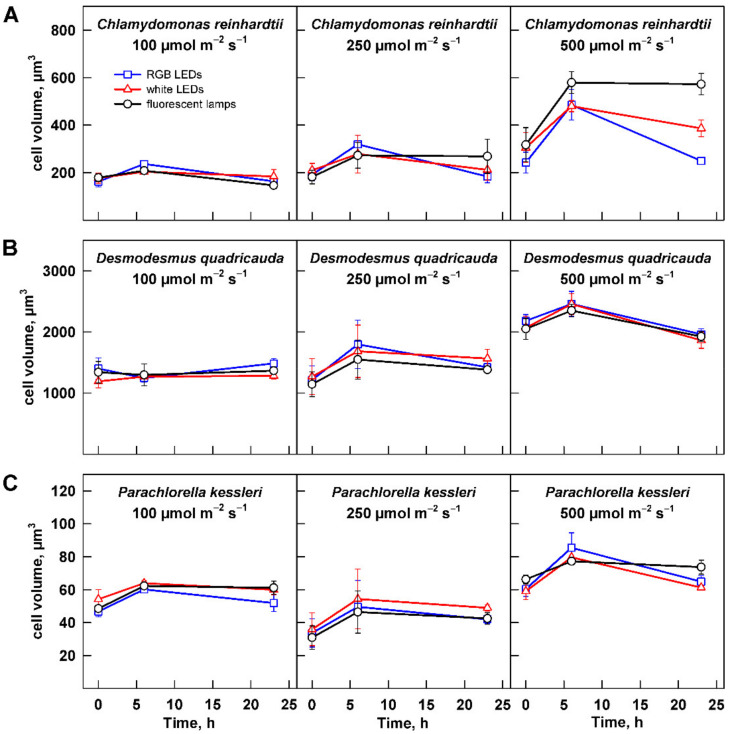
Changes in mean cell (coenobium) volume in the cultures grown at different light intensities. Cultures of (**A**) *Chlamydomonas reinhardtii*, (**B**) *Desmodesmus quadricauda*, and (**C**) *Parachlorella kessleri* were grown at incident light intensity 100, 250, and 500 µmol m^−2^ s^−1^ on different light sources: fluorescent lamps (black circles), RGB LEDs (blue squares), and white LEDs (red triangles). Data of three biological replicates are presented as means ± SE.

**Figure 6:**
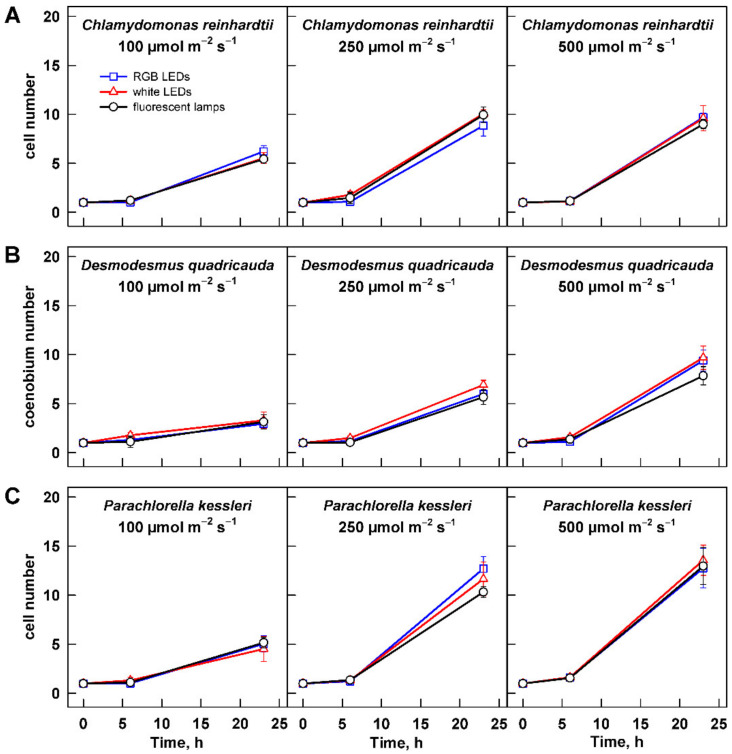
Changes in cell number in the cultures grown at different light intensities. Cultures of (**A**) *Chlamydomonas reinhardtii*, (**B**) *Desmodesmus quadricauda*, and (**C**) *Parachlorella kessleri* were grown at incident light intensity 100, 250, and 500 µmol m^−2^ s^−1^ on different light sources: fluorescent lamps (black circles), RGB LEDs (blue squares), and white LEDs (red triangles). Cell numbers were normalized to the initial values to allow direct comparison between treatments and organisms. Data of three biological replicates are presented as means ± SE.

The growth of the algae was further analyzed by assessing changes in cell number and cell size. Change in cell size is a direct measure of cell growth. For all three organisms, the maximum cell size was significantly increasing with light intensity (Figure 5, Table 2). The cell size generally increased from 0 to 6 h and decreased thereafter. The only exception to this rule was the culture of *C. reinhardtii* grown at 500 µmol m^−2^ s^−1^ (Figure 5A). In this treatment, the cells grown on fluorescent lamps did not decrease their size by 23 h (Figure 5A). This was caused by presence of a large percentage of dividing mother cells with daughter cells still organized in cell clusters. The clusters split shortly thereafter during the subsequent culture dilution so that the culture was made of single cells within one hour. This behavior was also reflected in the cell counts, where strikingly the cell numbers were lower in the cultures grown at 500 µmol m^−2^ s^−1^ compared with those grown at 250 µmol m^−2^ s^−1^ (Figure 6A). Except in this specific case, the analyses of cell numbers confirmed the outcomes of biomass analyses (compare Figure 4 and Figure 6). Furthermore, cell doubling time was calculated based on the changes in cell numbers across all the treatments (Table 3), and it again confirmed the behavior observed with changes in biomass. Doubling times were the longest for all the organisms grown at 100 µmol m^−2^ s^−1^; of these, *D. quadricauda* was again the slowest grower (Table 3). Doubling times for all three organisms were similar for cultures grown at 250 and 500 µmol m^−2^ s^−1^. This was in agreement with the results of biomass increase for *D. quadricauda* and *P. kessleri* but not *C. reinhardtii*. The difference is probably connected to the larger cell size at the higher light intensity (Figure 5A, Table 2), which would translate to higher biomass even at the same cell doubling time.

#### 3.2.3. Photosynthetic Pigments

**Figure 7:**
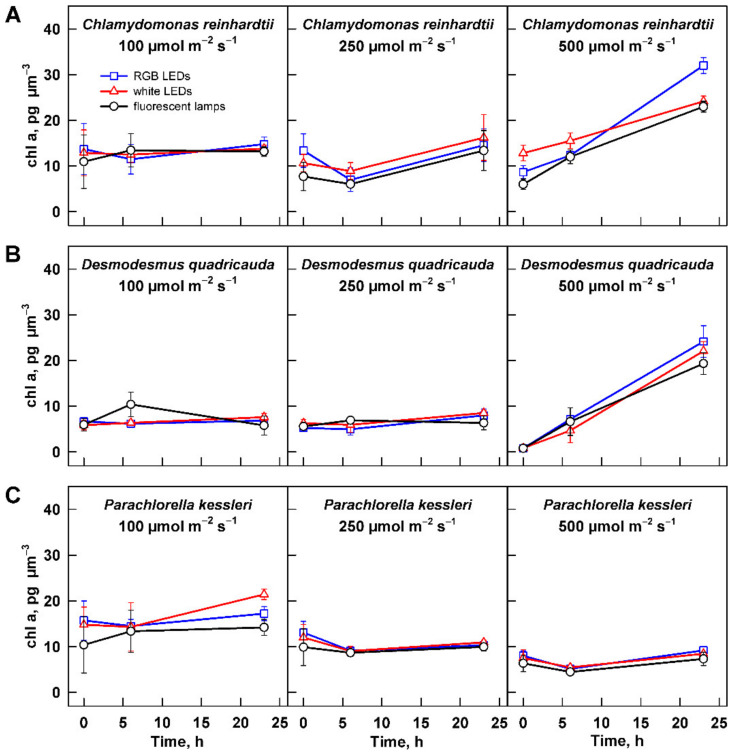
Changes in chlorophyll *a* content in the cultures grown at different light intensities. Cultures of (**A**) *Chlamydomonas reinhardtii*, (**B**) *Desmodesmus quadricauda*, and (**C**) *Parachlorella kessleri* were grown at incident light intensity 100, 250, and 500 µmol m^−2^ s^−1^ on different light sources: fluorescent lamps (black circles), RGB LEDs (blue squares), and white LEDs (red triangles). Chlorophyll content was normalized to the total volume of the culture at the particular time point to allow direct comparison between treatments and organisms. Data of three biological replicates are presented as means ± SE.

Light harvesting and utilization is affected by composition and concentration of photosynthetic pigments. The concentration of pigments in biomass is dependent on the biomass concentration and cell size, whilst the composition of the photosynthetic pigments is related to light intensity and quality. To allow for direct comparison among the three organisms and the treatments, the values of individual photosynthetic pigments were normalized to cell size of the population. The pigment concentration differed among the organisms, with *D. quadricauda* showing the lowest chlorophyll *a* and *b* concentrations (Figure 7 and Figure S1). The concentration of chlorophyll *a* and *b* were comparable between *C. reinhardtii* and *P. kessleri.* Furthermore, the organisms showed a species-specific response to increasing light intensity. The relationship was the simplest for *P. kessleri,* where the concentrations of chlorophyll *a* and *b* and carotenoids decreased with increasing light intensity (Figure 7C, Figures S1C and S2C). Cultures of *C. reinhardtii* and *D. quadricauda* maintained similar levels of photosynthetic pigments at 100 and 250 µmol m^−2^ s^−1^ (Figure 7A,B, Figures S1A,B and S2a,b), but their concentration increased with time of cultivation on the highest light intensity (Figure 7A,B, Figures S1A,B and S2a,b).

### 3.3. Effect of Light Quality

The second dimension of the presented series of experiments was to analyze a possible influence of light quality on cell growth and photosynthetic pigment composition. Throughout the data presented above (Figure 1, Figure 2, Figure 3, Figure 4, Figure 5, Figure 6 and Figure 7, Figures S1 and S2), there was no significant difference in the response of the culture to the light source used for cultivation. The only difference observed was in the cell size and cell number of *C. reinhardtii* cultures cultured at 500 µmol m^−2^ s^−1^ (Figure 5A and Figure 6A). However, this difference reflects only the faster or slower splitting of the divided mother cells into individual daughter cells. The difference is not present within 1 h, as the cultures show a similar distribution of cell size at 0 h of the experiment (Figure 5A and Figure 6A).

## 4. Discussion

Photoautotrophic growth of microalgae depends entirely on photosynthesis. Under natural growth conditions, these organisms use part of the sunlight spectrum from 400 to 700 nm (PAR) for photosynthesis [36]; the cultivation of microalgae in open systems also usually relies on this light source. However, in closed photobioreactors, the light is provided by an artificial light source with different intensity and light spectrum. The light spectrum—i.e., the composition and concentration of photons of different wavelength and energy—is crucial for photosynthesis and can affect not only the concentration but also the composition of biomass [23]. Recently, solid-state lighting technologies such as LEDs have become a preferred light source due to their low operating cost, long lifetime, and versatility of combining different wavelengths with narrow bandwidth [22,23]. There are a number of studies comparing the effects of different LED types with different light spectra on algal growth and productivity [7,19,20,22,23]. However, to our knowledge, no comprehensive comparison of algal growth with different white light sources has been conducted. However, since the spectral characteristics of the light sources differ (Figure 2), it is important to ensure that the choice of light source does not hamper algal growth. Another important light parameter that affects autotrophic growth is light intensity. Cultivation at low light intensity ensures slow growth. Improvement in growth can be achieved by optimizing growth conditions, including light intensity. Given the current increase in energy prices, it is important to determine the maximum light intensity that will promote growth to avoid the additional cost of an excessive light source.

In the current study, the growth of three algal species—two models with biotechnological potential, *C. reinhardtii* and *D. quadricauda*, and the biotechnologically important *P. kessleri*—was monitored. Each of the species showed a specific response to light intensity. Growth of *D. quadricauda* and *P. kessleri* was already saturated at 250 µmol m^−2^ s^−1^, and further increase in light intensity did not improve growth (Figure 3B,C, Table 1). In contrast, *C. reinhardtii* growth was not yet saturated, even at 500 µmol m^−2^ s^−1^ (Figure 3), although the 30% increase in growth rate from 250 to 500 µmol m^−2^ s^−1^ was less than the 150% increase from 100 to 250 µmol m^−2^ s^−1^ (Figure 3A, Table 1), indicating that these cultures were also close to saturation. All three species have a similar division pattern: multiple fission [37]. Organisms that divide by multiple fission are capable of dividing into 2, 4, 8, 16, or even more cells depending on growth conditions. This is achieved by uninterrupted growth in light as long as light is available. Cell division is postponed and is not allowed until the cells are moved to the dark or, if they grow in continuous light, until the maximum size of the mother cells is reached [37]. This behavior is reflected in the increase in maximum cell size with increasing light intensity in cultures of *C. reinhardtii* and *D. quadricauda* between 100 and 250 µmol m^−2^ s^−1^ and between 250 and 500 µmol m^−2^ s^−1^, respectively, and from 250 to 500 µmol m^−2^ s^−1^ in *P. kessleri* (Figure 4, Table 2). Such an increase in cell size is a specific response of organisms that divide by multiple fission and thus can respond to better growth conditions beyond a simple increase in cell number. Although the cell numbers produced were comparable between 250 and 500 µmol m^−2^ s^−1^ (Figure 5), the larger cell size is a mechanism that supports better growth in the next cell cycle, possibly leading to better productivity.

Apart from the changes in the number of cells produced and their size, the decreasing light intensity also affected the concentration of photosynthetic pigments in the cells. In *P. kessleri* cells, the concentration of all pigments (Figure 7, Figures S1 and S2) decreased with increasing light intensity. This is a typical response found not only in the genus *Chlorella* [7,38,39,40] but also in other green algae [41]. In contrast, both *C. reinhardtii* and *D. quadricauda* maintained similar concentrations of photosynthetic pigments at 100 and 250 µmol m^−2^ s^−1^ (Figure 7, Figures S1 and S2). This is surprising because *C. reinhardtii* and *Scenedesmus dimorphus*, a relative of *D. quadricauda*, had lower chlorophyll content at lower light intensity than at high light intensity. However, the conditions used in the two studies and in this work differ. In the experiments of Bonente et al., *C. reinhardtii* was acclimated to a lower light intensity of 60 µmol m^−2^ s^−1^ (compared with 200 µmol m^−2^ s^−1^ used in this study); it was also cultivated at a lower temperature of 21 °C [42]. Ferreira and colleagues also used a lower temperature when growing *Scenedesmus dimorphus* than in this study (25 °C versus 30 °C) [43]. More importantly, their high light intensity (125 µmol m^−2^ s^−1^) is close to the lowest light intensity used in this study. Our results suggest that the cells of *C. reinhardtii* and *D. quadricauda* have similar concentrations of photosynthetic pigments under optimal growth conditions, at least over a 2.5-fold range of light intensities. Interestingly, pigment concentrations in both organisms increased during cultivation at 500 µmol m^−2^ s^−1^ (Figure 7, Figures S1 and S2). This could reflect the decreasing mean light intensity absorbed by the cells with increasing biomass. The effect of increasing biomass could be more pronounced in the two species simply because of their larger cell size (Figure 4), which could affect light perception. Alternatively, the absence of this behavior in *P. kess-leri* could be a species-specific adaptation to growth at high cell and biomass density, which is typical for this species. The latter is supported by the higher cell number (13.6-fold increase) in the cultures of *P. kessleri* compared with an approximately 9.7-fold increase in *C. reinhardtii* and *D. quadricauda* (Figure 6). Of the three species, the concentration of photosynthetic pigments per cell was lowest in *D. quadricauda* (Figure 7, Figures S1 and S2), which may be related to the slowest growth of this organism of the three (Figure 3, Table 1).

The three white light sources used in this study—fluorescent lamps, RGB LEDs, and white LEDs—differed in their light spectra (Figure 2). Although the molar photon concentration was the same on PAR, each source provided different energy for photosynthesis. The absorption spectra of chlorophyll a, b, and carotenoids peak in the blue and red regions of PAR. Among the three light sources, RGB LED best matched the combined absorption spectra of chlorophyll a, b, and β-carotene [44]. Nevertheless, no difference in growth, maximum cell size, cell number, or photosynthetic pigment composition was observed among the three light sources. It is known that different light quality can affect cell size, as blue light induces the production of larger cells [26,27] but also growth rates and biomass composition [24]. Our results suggest an amazing versatility and robustness of the photosynthetic apparatus of the three algae, allowing them to grow comparably on the three different white light sources.

## 5. Conclusions

We describe the effect of different spectra of white light and different light intensity on the growth of three algal species: *Chlamydomonas reinhardtii*, *Desmodesmus quadricauda,* and *Parachlorella kessleri*. Increasing the light intensity improved the growth rates of all the species, with *Desmodesmus quadricauda* and *Parachlorella kessleri* reaching saturation light intensity at 250 µmol m^−2^ s^−1^. *Chlamydomonas reinhardtii* did not reach a saturation even at the highest incident light intensity of 500 µmol m^−2^ s^−1^. All species can divide into more than two daughter cells by multiple fission if growth conditions permit. Growth at increasing light intensity resulted in cell enlargement by factor of 2.5 in *Chlamydomonas reinhardtii*, 1.9 in *Desmodesmus quadricauda*, and only 1.3 in *Parachlorella kessleri.* The smaller increase in cell size in *Parachlorella kessleri* was compensated for by a 13.6-fold daily increase in cell number under optimal conditions, as compared with a 9.7-fold increase in *Chlamydomonas reinhardtii* and *Desmodesmus quadricauda*. *Desmodesmus quadricauda* grew the slowest of the organisms tested, which may be due to the lowest concentration of photosynthetic pigments in its cells. The concentration of chlorophyll *a* and *b* as well as carotenoids decreased in *Parachlorella kessleri* with increasing light intensity. In contrast, it remained at similar levels in *Chlamydomonas reinhardtii* and *Desmodesmus quadricauda* at both 100 and 250 µmol m^−2^ s^−1^. None of the three white light sources—fluorescent lamps, RGB LEDs, and white LEDs—altered the growth or affected photosynthetic pigment concentration and composition. This indicates the versatility and robustness of algal photosynthesis. Our results show species-specific response of three biotechnologically interesting species to increasing light intensity. Furthermore, they show that white light source has no effect on their growth in the exponential phase, although there might be some effect in the stationary phase.

## Figures and Tables

**Table 1:** Maximum optical density reached by cultures of *Chlamydomonas reinhardtii*, *Desmodesmus quadricauda*, and *Parachlorella kessleri* grown at different incident light intensities. Fold increase in optical density between 0 and 23 h was calculated from the values of absorbance at 750 nm. Light source is stated above the columns. Data of three biological replicates are presented as means ± SE. Values were statistically significantly different between *Desmodesmus quadricauda* and both *Chlamydomonas reinhardtii* and *Parachlorella kessleri* but not between the latter two organisms; they were statistically significantly different between all light intensities in *Chlamydomonas reinhardtii* and *Desmodesmus quadricauda* but only between 100 and 250 µmol m^−2^ s^−1^ (and 100 and 500 µmol m^−2^ s^−1^) in *Parachlorella kessleri* (two-way ANOVA, Tukey’s HSD test, *p* < 0.05, *n* = 3).

	Incident Light Intensity (µmol m^−2^ s^−1^)		
Organism	100	250	500
	Fluorescent Lamps		
*Chlamydomonas reinhardtii*	4.68 ± 0.36	7.18 ± 0.28	8.12 ± 0.83
*Desmodesmus quadricauda*	3.19 ± 0.28	6.4 ± 0.1	7.24 ± 0.22
*Parachlorella kessleri*	4.96 ± 0.29	8.26 ± 0.46	8.35 ± 0.17
	RGB LED		
*Chlamydomonas reinhardtii*	4.79 ± 0.86	7.69 ± 0.69	9.29 ± 0.37
*Desmodesmus quadricauda*	3.63 ± 0.28	5.94 ± 0.39	6.94 ± 0.52
*Parachlorella kessleri*	4.3 ± 0.23	7.96 ± 0.65	8.52 ± 0.28
	White LED		
*Chlamydomonas reinhardtii*	4.52 ± 0.57	7.37 ± 0.41	9.51 ± 0.92
*Desmodesmus quadricauda*	3.21 ± 0.29	6.65 ± 0.28	6.68 ± 0.52
*Parachlorella kessleri*	4.4 ± 0.18	8.1 ± 0.8	8.83 ± 0.17

**Table 2:** Maximum cell size (µm^3^) reached in cultures of *Chlamydomonas reinhardtii*, *Desmodesmus quadricauda*, and *Parachlorella kessleri* grown at different incident light intensities. The data are derived from 6 h of experiment when cells of all the treatments reached their maximum cell size. Light source is stated above the columns. Data of three biological replicates are presented as means ± SE. Cell sizes were statistically significantly different both within the organisms and within different light intensities (two-way ANOVA, Tukey´s HSD test, *p* < 0.05, *n* = 3).

	Incident Light Intensity (µmol m^−2^ s^−1^)		
Organism	100	250	500
	Fluorescent lamps		
*Chlamydomonas reinhardtii*	208 ± 12	323 ± 21	579 ± 46
*Desmodesmus quadricauda*	1297 ± 180	1964 ± 169	2349 ± 1032
*Parachlorella kessleri*	62 ± 1	46 ± 4	77 ± 2
	RGB LED		
*Chlamydomonas reinhardtii*	236 ± 11	355 ± 14	485 ± 64
*Desmodesmus quadricauda*	1244 ± 67	2218 ± 132	2459 ± 204
*Parachlorella kessleri*	60 ± 2	50 ± 2	85 ± 9
	White LED		
*Chlamydomonas reinhardtii*	215 ± 11	393 ± 32	613 ± 24
*Desmodesmus quadricauda*	1267 ± 32	2209 ± 141	2456 ± 172
*Parachlorella kessleri*	64 ± 1	54 ± 5	80 ± 1

**Table 3:** Cell doubling times (h) of cultures of *Chlamydomonas reinhardtii*, *Desmodesmus quadricauda*, and *Parachlorella kessleri* grown at different incident light intensities. Data were calculated from the interval from 0 to 23 h of cultivation. Light source is stated above the columns. Data of three biological replicates are presented as means ± SE. Values were statistically significantly different between *Desmodesmus quadricauda* and both *Chlamydomonas reinhardtii* and *Parachlorella kessleri* but not between the latter two organisms; they were statistically significantly different for all organisms between 100 and 250 µmol m^−2^ s^−1^ as well as 100 and 500 µmol m^−2^ s^−1^ but not between 250 and 500 µmol m^−2^ s^−1^ (two-way ANOVA, Tukey´s HSD test, *p* < 0.05, *n* = 3).

	Incident Light Intensity (µmol m^−2^ s^−1^)		
Organism	100	250	500
	Fluorescent lamps		
*Chlamydomonas reinhardtii*	9.44 ± 0.16	6.98 ± 0.25	7.28 ± 0.18
*Desmodesmus quadricauda*	12.68 ± 0.92	9.43 ± 0.83	7.85 ± 0.49
*Parachlorella kessleri*	9.89 ± 0.73	6.84 ± 0.16	6.33 ± 0.41
	RGB LED		
*Chlamydomonas reinhardtii*	8.82 ± 0.5	7.4 ± 0.37	7.03 ± 0.13
*Desmodesmus quadricauda*	12.36 ± 0.47	8.57 ± 0.12	7.19 ± 0.34
*Parachlorella kessleri*	9.27 ± 0.29	6.31 ± 0.26	6.39 ± 0.44
	White LED		
*Chlamydomonas reinhardtii*	9.43 ± 0.56	6.91 ± 0.11	7.17 ± 0.49
*Desmodesmus quadricauda*	12.61 ± 0.55	8.28 ± 0.28	7.12 ± 0.44
*Parachlorella kessleri*	9.11 ± 0.41	6.62 ± 0.47	6.16 ± 0.25

## Data Availability

All data presented in this study are available within this article or Supplementary Materials. There are no special databases associated with this manuscript.

## Supplementary Materials

The following supporting information can be downloaded at: https://www.mdpi.com/article/10.3390/cells11081293/s1, Figure S1: Changes in chlorophyll *b* content in the cultures grown at different light intensities, Figure S2: Changes in carotenoid content in the cultures grown at different light intensities.
