# Supplementary material for: The Effect of Variable Light Source and Light Intensity on the Growth of Three Algal Species
Source: Cells. 2022 Apr 11;11(8):1293. doi: 10.3390/cells11081293 (PMC9028354; doi:10.3390/cells11081293)
Supplement: Supplementary file 1 [file cells-11-01293-s001.zip › cells-1662033-supplementary.pdf]

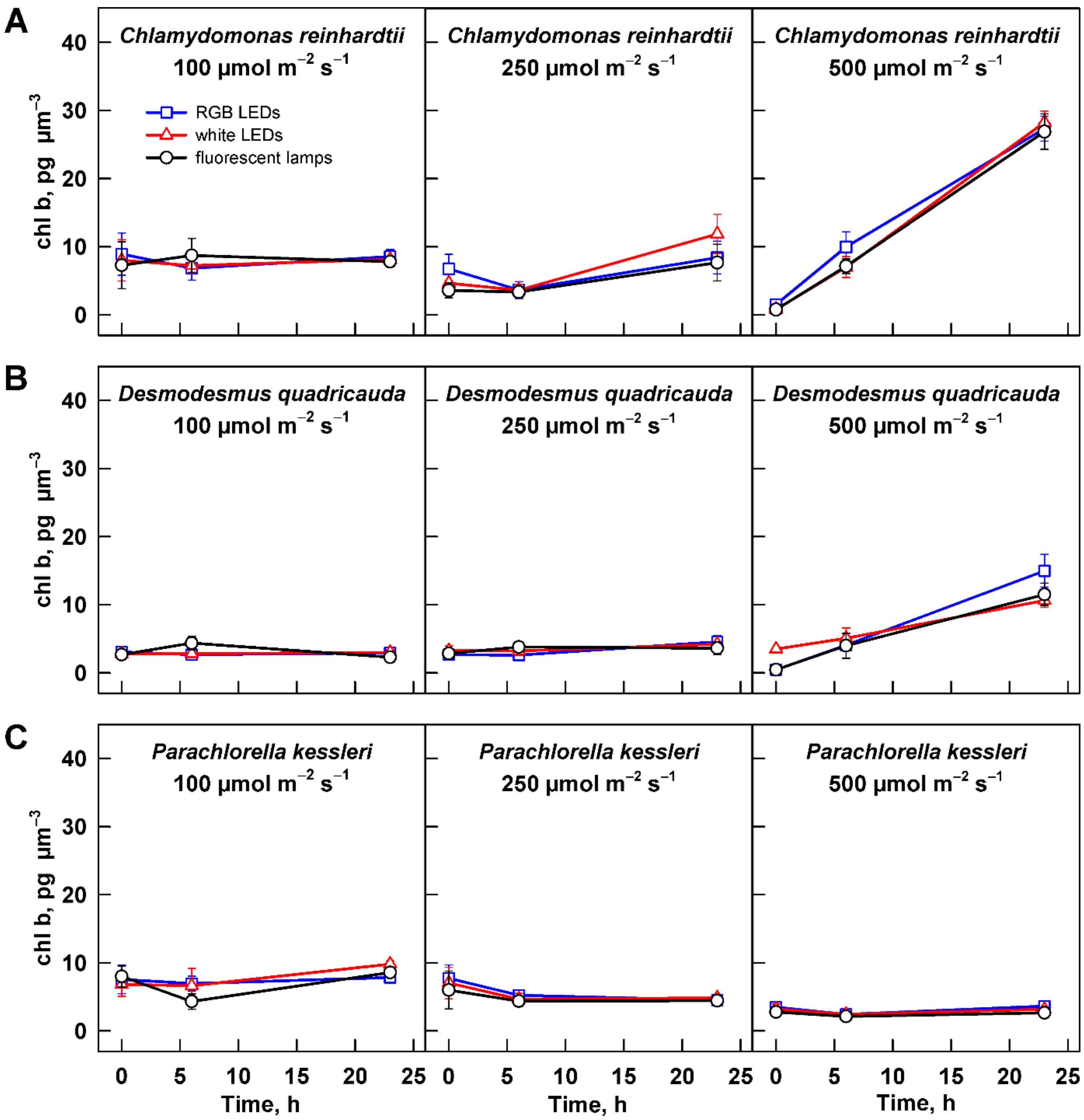

Figure S1 Changes in chlorophyll *b* content in the cultures grown at different light intensities. Cultures of (**A**) *Chlamydomonas reinhardtii*, (**B**) *Desmodesmus quadricauda*, (**C**) *Parachlorella kessleri* were grown at incident light intensity 100, 250 and 500 μmol m^{-2} s^{-1} on different light sources: fluorescent lamps (black circles), RGB LEDs (blue squares) and white LEDs (red triangles). Chlorophyll content was normalized to the total volume of the culture at the particular time-point to allow direct comparison between treatments and organisms. Data of three biological replicates are presented as means ± SE.

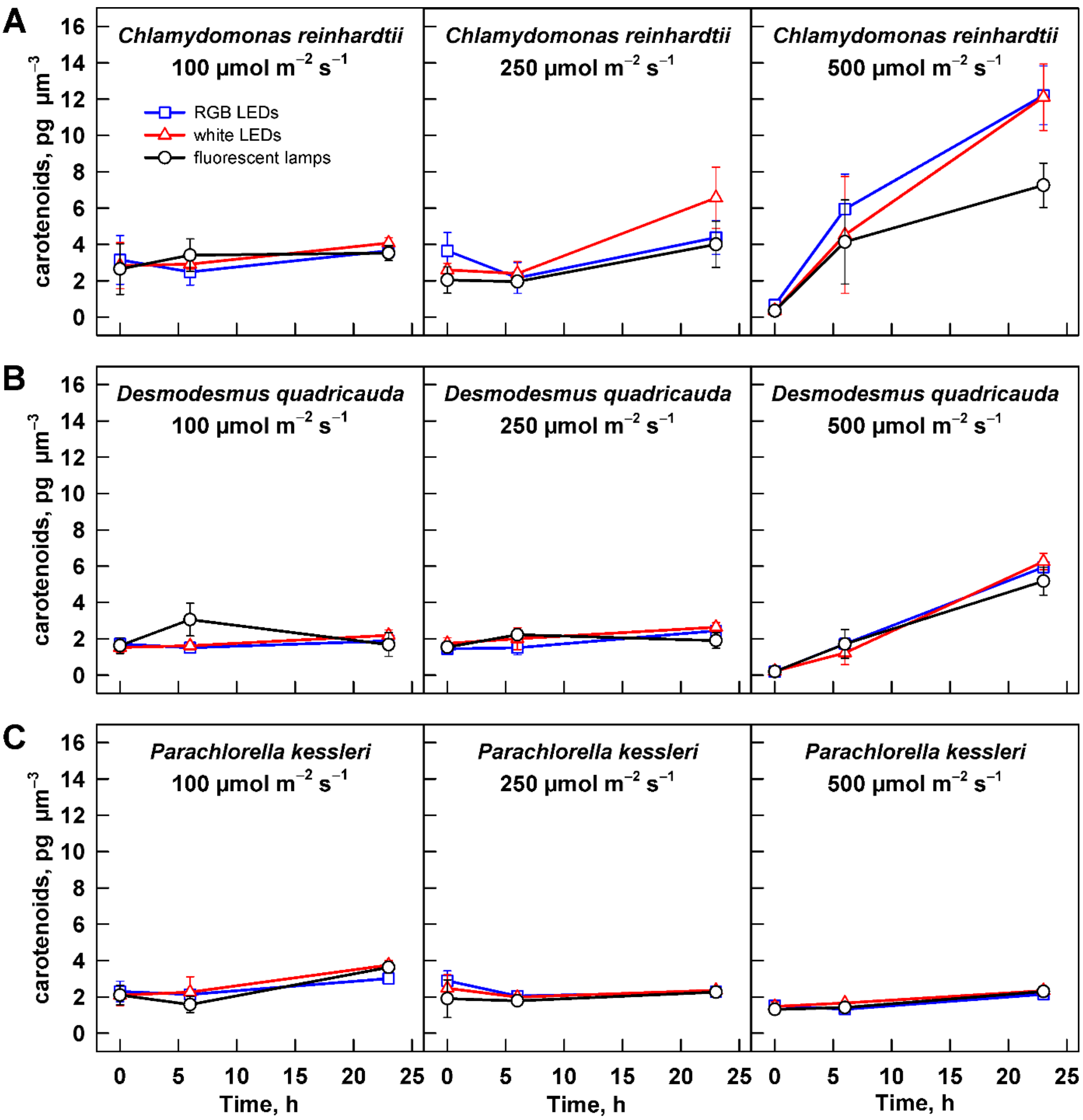

Figure S2 Changes in carotenoid content in the cultures grown at different light intensities. Cultures of (**A**) *Chlamydomonas reinhardtii*, (**B**) *Desmodesmus quadricauda*, (**C**) *Parachlorella kessleri* were grown at incident light intensity 100, 250 and 500 µmol m^{-2} s^{-1} on different light sources: fluorescent lamps (black circles), RGB LEDs (blue squares) and white LEDs (red triangles). Carotenoid content was normalized to the total volume of the culture at the particular time-point to allow direct comparison between treatments and organisms. Data of three biological replicates are presented as means ± SE.
